# Supplementary material for: Assessing the Prognostic Value of Cytoplasmic and Stromal Caveolin-1 in Early Triple-Negative Breast Cancer Undergoing Neoadjuvant Chemotherapy
Source: Int J Mol Sci. 2024 Nov 14;25(22):12241. doi: 10.3390/ijms252212241 (PMC11594706; doi:10.3390/ijms252212241)
Supplement: Supplementary file 1 [file ijms-25-12241-s001.zip › Suplementary Table S1 Correlation of CAV1 levels and patients’ characteristics.pdf]

Supplementary Table S1. Correlation of CAV1 levels and patients' characteristics; p-value calculated using Mann-Whitney U test, Fisher's test, T-Student test, or Chi-Square test, with significance set at 0.05.

Variable	Patients +cCAV1 (N=6)	Patients -cCAV1 (N=52)	p	Patients +sCAV1 (N=24)	Patients -sCAV1 (N=33)	p
**Age** (y), Mean (min-max)	59,5 (36-82)	51,6 (25-83)	0,12	53,8 (31-83)	52,1 (25-81)	0,35
**Menopausal status, n (%)**			0,99			0,29
Yes	3 (50%)	28 (54%)		15 (62%)	15 (48%)	
Not	3 (50%)	24 (46%)		9 (38%)	17 (52%)	
**Clinical Stage, n (%)**			0,33			0,38
II	3 (50%)	39 (75%)		**19 (79%)**	22 (67%)	
III	3 **(50%)**	13 **(25%)**		5 (21%)	11 (33%)	
**Nodal status, n (%)**			0,99			0,48
Negative	2 (33%)	21 (40%)		8 (33,3)	14 (42,4)	
Positive	4 (67%)	31 (60%)		16 (66,7)	19 (57,6)	
**Ki67 mean (min-max)**	63,8 (20-90)	69,3 (25-95)	0,53	63,9 (25-90)	**73,6 (20-95)**	0,07
**Histological grade, n (%)**			0,57			0,71
II	0 (0)	10 (19%)		4 (16%)	4 (12%)	
III	**6 (100%)**	42 (81%)		20 (84%)	29 (88%)	
**TILS mean (standar desv)**	-	-	-	11,43 (9,9)	33 (31,3)	0,06
**Chemotherapy, n (%)**			0,09			0,53
NP - EC	3 (50%)	43 (83%)		20 (83%)	25 (76%)	
NP	3 (50%)	9 (17%)		4 (17%)	8 (24%)	
**Adjuvant Radiotherapy, n (%)**			0,23			0,47
Yes	4 (33%)	45 (87%)		19 (79%)	29 (88%)	
Not	2 (67%)	7 (13%)		5 (21%)	4 (22%)	

Abbreviations: TILS tumor-infiltrating lymphocytes; NP nab-paclitaxel; EC epirrubicine and cyclophosphamide
