# Supplementary figures and images for: Assessing the Prognostic Value of Cytoplasmic and Stromal Caveolin-1 in Early Triple-Negative Breast Cancer Undergoing Neoadjuvant Chemotherapy
Source: Int J Mol Sci. 2024 Nov 14;25(22):12241. doi: 10.3390/ijms252212241 (PMC11594706; doi:10.3390/ijms252212241)

## Slide 1
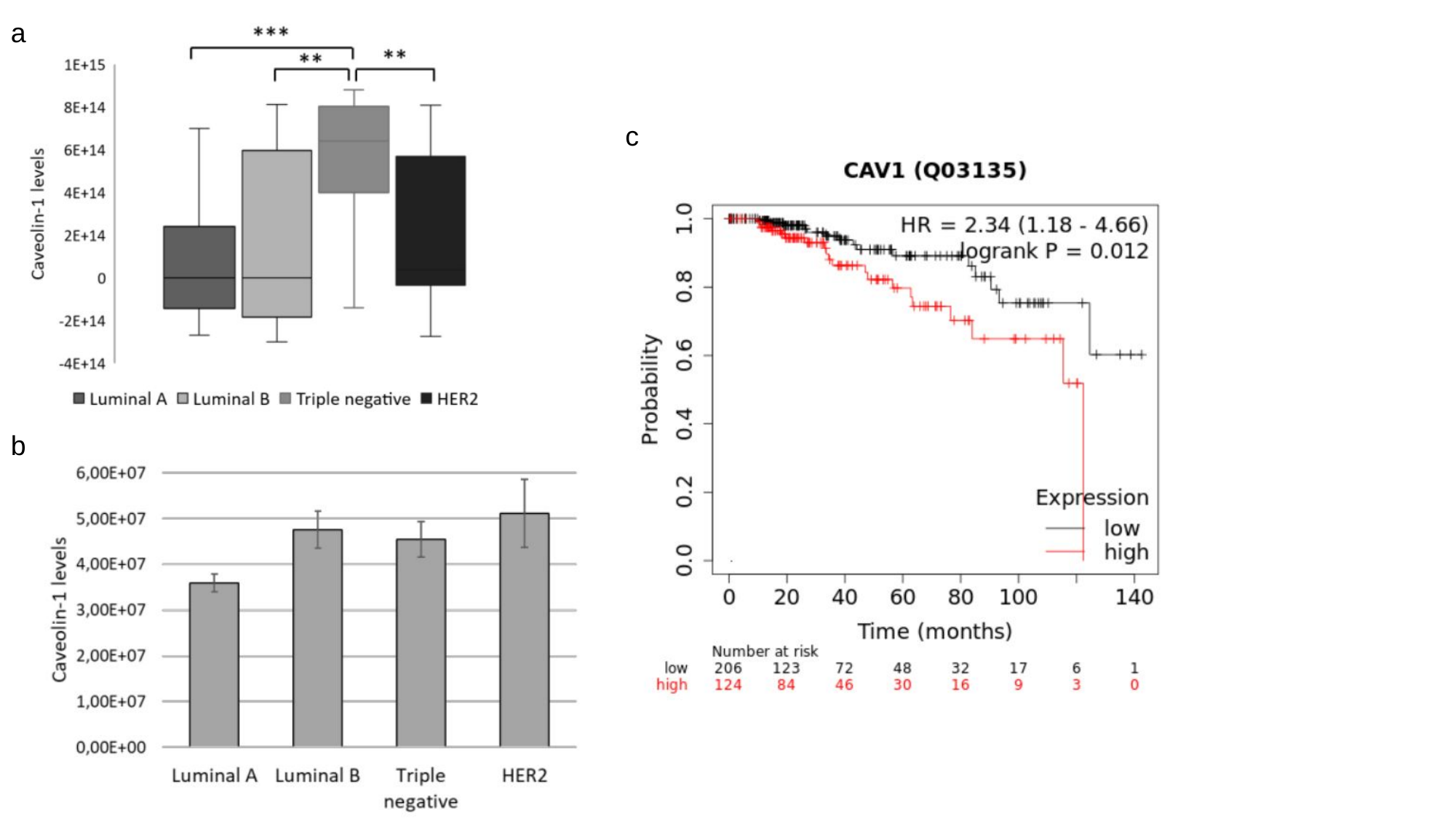

a
c
b

Supplement: Supplementary file 1 [file ijms-25-12241-s001.zip › Suplementary Figure S1 in silico.pptx]
